# Hypertension with unsatisfactory sleep health (HUSH): study protocol for a randomized controlled trial

**DOI:** 10.1186/s13063-017-2001-9

**Published:** 2017-06-06

**Authors:** Jessica C. Levenson, Bruce L. Rollman, Lee M. Ritterband, Patrick J. Strollo, Kenneth J. Smith, Jonathan G. Yabes, Charity G. Moore, Allison G. Harvey, Daniel J. Buysse

**Affiliations:** 1 Department of Psychiatry, University of Pittsburgh School of Medicine, 3811 O’Hara Street, E-1123 TDH, Pittsburgh, PA 15213 USA; 2 Division of General Internal Medicine, Center for Behavioral Health and Smart Technology, University of Pittsburgh School of Medicine, Pittsburgh, PA USA; 3 Department of Psychiatry and Neurobehavioral Sciences and Center for Behavioral Health and Technology, University of Virginia School of Medicine, Charlottesville, VA USA; 4 VA Pittsburgh Health System, Pittsburgh, PA USA; 5 Department of Biostatistics, University of Pittsburgh School of Medicine, Pittsburgh, PA USA; 6 Carolinas HealthCare System, Charlotte, NC USA; 7 Department of Psychology, University of California, Berkeley, CA USA

**Keywords:** Sleep, Hypertension, Technology, Blood pressure

## Abstract

**Background:**

Insomnia is common in primary care medical practices. Although behavioral treatments for insomnia are safe, efficacious, and recommended in practice guidelines, they are not widely-available, and their effects on comorbid medical conditions remain uncertain. We are conducting a pragmatic clinical trial to test the efficacy of two cognitive behavioral treatments for insomnia (Brief Behavioral Treatment for Insomnia (BBTI) and Sleep Healthy Using the Internet (SHUTi)) versus an enhanced usual care condition (EUC).

**Methods/Design:**

The study is a three-arm, parallel group, randomized controlled trial. Participants include 625 adults with hypertension and insomnia, recruited via electronic health records from primary care practices affiliated with a large academic medical center. After screening and baseline assessments, participants are randomized to treatment. BBTI is delivered individually with a live therapist via web-interface/telehealth sessions, while SHUTi is a self-guided, automated, interactive, web-based form of cognitive behavioral therapy for insomnia. Participants in EUC receive an individualized sleep report, educational resources, and an online educational video. Treatment outcomes are measured at 9 weeks, 6 months, and 12 months. The primary outcome is patient-reported sleep disturbances. Secondary outcomes include other self-reported sleep measures, home blood pressure, body mass index, quality of life, health functioning, healthcare utilization, and side effects.

**Discussion:**

This randomized clinical trial compares two efficacious insomnia interventions to EUC, and provides a cost-effective and efficient examination of their similarities and differences. The pragmatic orientation of this trial may impact sleep treatment delivery in real world clinical settings and advance the dissemination and implementation of behavioral sleep interventions.

**Trial registration:**

ClinicalTrials.gov (Identifier: NCT02508129; Date Registered: July 21, 2015).

**Electronic supplementary material:**

The online version of this article (doi:10.1186/s13063-017-2001-9) contains supplementary material, which is available to authorized users.

## Background

Insomnia is characterized by self-reported difficulty in falling asleep, staying asleep or poor quality sleep, adequate opportunity for sleep, and daytime symptoms such as fatigue, irritability and impaired concentration [1]. Insomnia is prevalent, persistent, and costly. An estimated 10–15% of adults have insomnia disorder and 50% have subsyndromal insomnia symptoms each year [2], while symptoms persist for 3 or more years in at least 40% of individuals [3]. Additionally, insomnia is associated with a range of adverse health outcomes, including impaired health-related quality of life and increased risk for depression, suicide, hypertension (HTN), cardiovascular disease, and mortality [1].

Efficacious pharmacologic treatments for insomnia are available [4–6]. However, concerns remain regarding the adverse effects of benzodiazepine receptor agonist hypnotics, including sedation, anterograde amnesia, anxiety, impaired balance, increased falls and hip fractures, motor vehicle accidents, complex sleep-related behaviors, and risk for dependence and abuse [7–10]. Given these potential disadvantages, many patients prefer non-drug treatments, such as cognitive-behavioral treatment [11, 12]. The most widely investigated treatment is multi-modal Cognitive-Behavioral Treatment for Insomnia (CBT-I) [13–16], a 6–8 session, manualized, personalized intervention that combines several behavioral and cognitive strategies [17]. CBT-I is typically delivered by a trained sleep therapist in individual sessions and reliably improves both the general and sleep-specific symptoms of insomnia in individuals with and without medical and psychiatric comorbidities [13, 18–22], with durable effects over follow-up intervals of up to 3 years [23, 24] and no major adverse effects other than transient sleepiness if sleep is restricted too severely [25]. Recent clinical practice guidelines recommend CBT-I as the first-line treatment for insomnia [26]. However, CBT-I is not widely-available in primary care practices. The approximately 300 certified behavioral sleep specialists fall far short of the demand created by up to 30 million US adults with insomnia, and few of these specialists work in primary care settings [27]. Moreover, at US$40 per hour, an average course of CBT-I with a clinical psychologist costs US$240–$320 per patient, not including other related expenses such as lost work for appointments and travel time [28]. Alternate forms of CBT-I, such as group treatment, Internet-based treatment [29, 30], telephone consultation [31], and brief versions [32, 33] attest to its robust efficacy and potential for dissemination [34]. To the best of our knowledge, the only prior work to specifically focus on CBT-I in primary care involved nurse therapists treating insomnia in small group settings in the UK [35]. While brief, therapist-guided insomnia treatment appears efficacious, few studies have examined this modality [33, 36], and none in primary care settings.

Insomnia is frequently co-morbid with other psychiatric and medical conditions. In particular, insomnia is cross-sectionally associated with HTN [37, 38], with the risk of insomnia being 1.5–3.18 times higher among adults with HTN as compared to those without [39, 40]. Moreover, both insomnia and HTN are associated with increased risk of cardiovascular death [41–43], increasing the potential for negative health outcomes through mechanisms such as altered sympathovagal balance [44, 45] and increased inflammation [46, 47]. Moreover, insomnia and HTN are among the most common conditions in primary care practices, indeed insomnia occurs in 10–26% of patients [48–50] and HTN – the single most common condition seen in primary care [42] – occurs in 34–75%, depending on patient body mass index (BMI) and age [6, 51]. Previous studies have shown CBT-I to be efficacious for improving comorbid conditions such as depression [52] and pain [53], but none has focused on blood pressure (BP) control. Thus, HTN is an ideal secondary target for insomnia treatment in primary care settings.

In this trial, two innovative but distinct CBT-I-based interventions for insomnia (Brief Behavioral Treatment of Insomnia (BBTI) and Sleep Healthy Using the Internet (SHUTi)) are evaluated and compared to enhanced usual care (EUC) for insomnia among participants with insomnia and HTN. Which CBT-I adaptation is best suited to primary care settings remains an open question.

The aims of the Hypertension with Unsatisfactory Sleep Health (HUSH) clinical trial are to compare (1) the three interventions on patient-reported symptoms at 9 weeks, 6 months, and 12 months; (2) interventions on health indicators including self-report, home blood pressure monitoring (HBPM), and electronic health record (EHR) measures at 6 and 12 months; (3) patient and provider-level satisfaction among the interventions; and (4) BBTI and CBT-I on each outcome domain and intervention adherence or drop-outs (exploratory aim). We hypothesize that BBTI and SHUTi will be superior to EUC on patient-reported symptoms (sleep, depression, anxiety, and fatigue) at 9 weeks, 6 months, and 12 months, and on health indicators (HBPM, hypnotic use, quality of life, and healthcare utilization and costs) at 6 and 12 months. We also hypothesize that BBTI and SHUTi will have superior measures of patient and provider level satisfaction compared to EUC. Our trial includes those patients who are most representative of insomnia disorder (those with comorbid conditions such as HTN), in locations where most people seek treatment for insomnia (primary care physician offices), using outcomes that matter to patients and providers (self-reported symptoms, BP).

## Methods

### Overview of study design

**Fig. 1:**
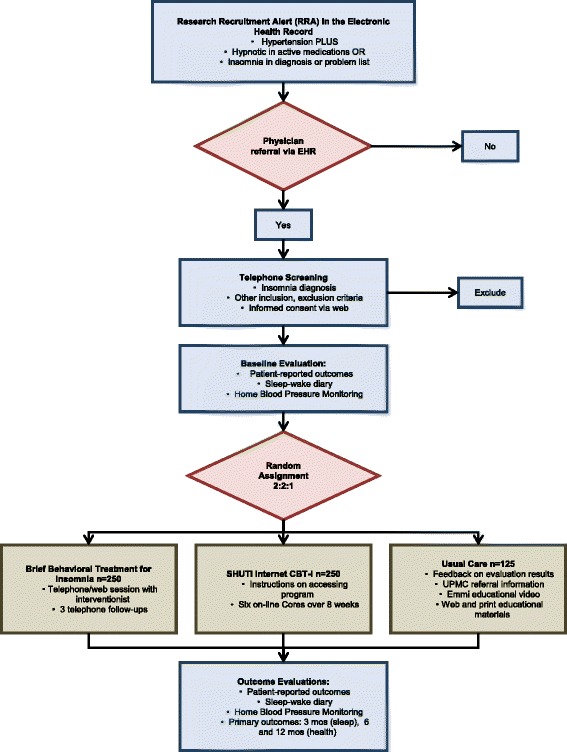
Study flow diagram

HUSH is a pragmatic, patient-centered, parallel-group, randomized, controlled trial comparing two CBT-I-based interventions for insomnia to EUC. The study has been approved by the University of Pittsburgh Institutional Review Board (last renewed on May 2, 2016, REN16040175/PRO14070337) and is registered with ClinicalTrials.gov (Identifier: NCT02508129; last updated January 2016). Study reporting adheres to Standard Protocol Items: Recommendations for Interventional Trials Reporting (Additional file 1) [54, 55]. Participants meeting study eligibility criteria are enrolled after completing initial screening and signing informed consent (Fig. 1). Following baseline assessments, they are randomly assigned to the BBTI, SHUTi, or EUC condition. Participants complete follow-up assessments at three time points (9 weeks, 6 months, 12 months), after completing the intervention phase. Trial conduct is overseen by three committees, namely a study investigators’ team, which provides guidance and makes key decisions about the conduct and implementation of the study as a whole; a study protocol management team, which addresses specific challenges in protocol procedures and data management; and an external data and safety monitoring board (DSMB; described below).

### Participants

The study will enroll a total of 625 men and women ages 18 and over with a diagnosis of HTN, and either hypnotic use or insomnia diagnosis (INS). Because this study is designed to recruit a sample of primary care patients with INS plus HTN and other typical co-morbidities, our eligibility criteria are intentionally broad. Participants from all racial and ethnic groups who plan to remain under the care of their current University of Pittsburgh Medical Center (UPMC) provider for at least 1 year are enrolled. Inclusion and exclusion criteria and their method of ascertainment are summarized in Table 1. Because all participant contact, measures, and interventions occur via telephone or Internet, participants are required to have reliable Internet access. Participants are excluded if they have current, severe, untreated sleep disorders other than insomnia. We do not exclude participants for using medications known to affect sleep or wake function (e.g., caffeine, alcohol, hypnotics, benzodiazepines, antidepressants, anxiolytics, antipsychotics, decongestants and sedating antihistamines, beta blockers, corticosteroids), since they are common among “real-life” insomnia patients. Educational components of the interventions are, in part, targeted at the use of these substances and medications. Participants’ use of these medications is monitored via sleep-wake diaries and medication lists in the EHR.

**Table 1:** Eligibility criteria

Criterion	Method
Inclusion criteria	
Age 18–75 years	Interview
Hypertension diagnosis	RRA; Diagnosis code 401.9
Prescription within a 12-month period of the following: zolpidem, zaleplon, eszopiclone, triazolam, or temazepam^a^	RRA; Name-brand or generic
Prescription within a 12-month period, with instructions to take at bedtime, of the following: trazodone, doxepin, amitriptyline, lorazepam, clonazepam^a^	RRA; Name-brand or generic
DSM-5 insomnia diagnosis^a^	Diagnosis codes 307.42, 307.47, or 780.52, or listed in chart’s ‘problem list’; verified with structured interview
At least moderate insomnia severity: Score ≥ 8 on Insomnia Severity Index [59]	Telephone questionnaire
Telephone, email address, reliable Internet access	Interview
Stable medical, psychiatric condition; no hospitalizations in 3 months	Interview
Exclusion criteria	
Untreated current major depression (PHQ-9 score ≥ 10; GAD-7 score ≥ 10); patients using stable (3 months) medication or psychological treatment are eligible	Telephone PHQ-9 [61], GAD-7 [62]; Interview
Bipolar disorder	RRA with diagnosis code 296.8, or Interview
Dementia, probable dementia, or mild cognitive impairment	RRA with diagnosis codes 290.XX-294.xx or 331.83, and Telephone Montreal Cognitive Assessment [93]
Substance use disorders within the past 3 months	Diagnosis codes 291.XX, 292.XX, 303.XX-305.XX, and NIDA Quick Screen [63]
Schizophrenia or psychotic disorder	RRA with diagnosis codes 293.XX-298.XX and Interview
Treatment with lithium or antipsychotic medication	RRA and electronic health record review; Indicates likely diagnosis of bipolar disorder or psychotic disorder
Active suicidal ideation or psychosis	DSM-5 Dimensional Symptom Assessment [94]
Severe obstructive sleep apnea (Apnea Hypopnea Index > 50 or oxyhemoglobin saturation < 85% for > 5% of the night)	Screening assessment for sleep disorders; ApneaLink Plus
Untreated and severe insufficient sleep syndrome, delayed sleep phase syndrome (habitual bedtimes later than 2:00 a.m., habitual waketimes later than 9:00 a.m.), narcolepsy, severe restless legs syndrome, and current night shift work (i.e., any work occurring between the hours of midnight and 6:00 a.m.)	Screening assessment for sleep disorders
Plans to move or leave present source of care during the following year	Interview
Non-English speaking, illiterate, or sensory deficits	Interview

^a^Participants must meet one of these criteria to be eligible for inclusion

*DSM-5* Diagnostic and Statistical Manual of Mental Disorders (5th edition), *GAD-7* Generalized Anxiety Disorder 7-item scale, *PHQ-9* Patient Health Questionnaire (9 item), *RRA* Research Recruitment Alert

### Recruitment

Participants are recruited via alerts triggered in the EpicCare EHR from approximately 20 of the 120 primary care practices affiliated with the UPMC, Pittsburgh, PA, USA. We maintain engagement with practices through yearly practice visits, quarterly physician newsletters, and reporting patient progress through the protocol to referring physicians. To serve as additional reminders, at regular intervals we offer study-branded materials, posters, brochures, and pens to participating practices.

**Fig. 2:**
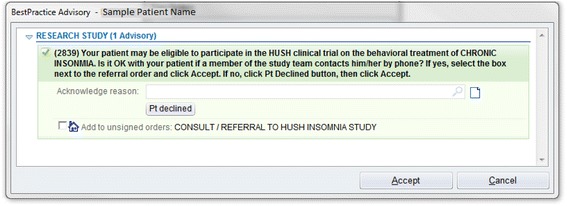
Example of research recruitment alert

Recruitment is conducted using physician alerts in the EpicCare EHR. At the time of the patient encounter, physicians in the participating primary care practices receive a Health Insurance Portability and Accountability Act-compliant Research Recruitment Alert (RRA; Fig. 2) in the EHR, triggered by relevant patient characteristics. A list of specific ICD codes that trigger the RRA (and exclusion criteria codes that cause an alert not to be fired) are available in Table 1. The physician then asks the patient if they would like to consider participating in our study. Upon obtaining the patients’ initial assent, physicians refer eligible patients to the study by clicking a “yes” or “no” button in the EHR. A “yes” response triggers an RRA with the patient’s basic information to be transmitted to the study team, including name, contact information, date of birth, primary care physician name, and allergies, among other basic patient information. Upon receiving the RRA, an automatically-generated email is sent from the study team to the patient, inviting them to complete an online screening, or to arrange a telephone screening with a study staff member. If the patient does not have an email address or does not respond to email, the patient is contacted via telephone. Physicians may also initiate a patient referral directly to the trial via the EHR, permitting referrals outside of office visits and for patients who may qualify, but whose EHR did not trigger a RRA.

**Fig. 3:**
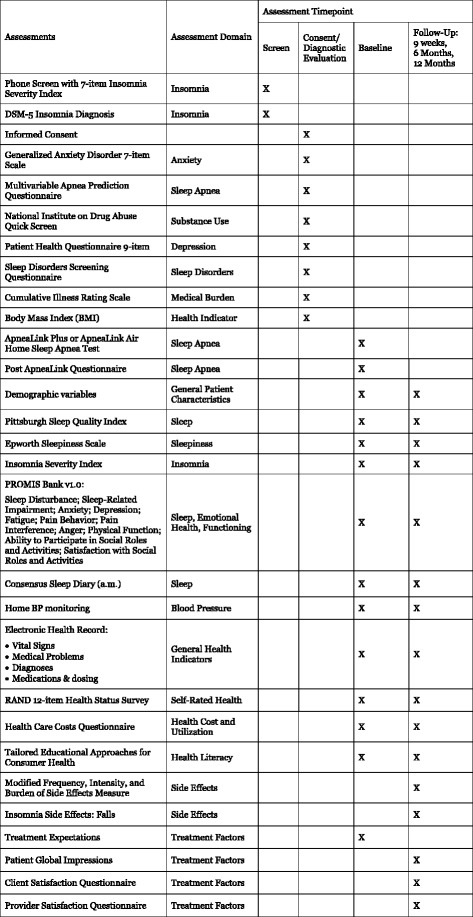
Study procedures and assessment grid

### Screening and consent

Potential participants complete initial study screening online, or via an identical telephone interview. The screening provides a brief description of the research study, interventions, and assessment procedures. If the patient is interested and elects to complete the online screening, they answer several questions related to demographic information, sleep characteristics, symptoms of insomnia, medical history, and computer and Internet access, using the instruments described above.

If the patient is initially eligible to proceed based on this information and expresses interest, a phone appointment is scheduled to review the study consent form and to conduct additional eligibility assessments. The participant is emailed the consent form and the URL to the study website. During the phone appointment, the patient logs in and reviews the online consent form with a staff member. The patient has the opportunity to ask questions and clarifications before electronically signing the consent. Study staff then review additional study criteria (Fig. 3) with the participant to ensure eligibility, entering these data directly via the web interface. The absence of exclusionary diagnoses (e.g., bipolar disorder) is verified by study staff after the interview, by reviewing the patient’s problem list in the EHR. Additional medical information is also extracted from the patient’s EHR. We reduce the risk of receiving unnecessary information from the patient’s chart through the use of an intermediary, the Center for Assistance in Research using e-Record, a joint program of the University of Pittsburgh, and UPMC. Medical information is identified only by participants’ UPMC medical record number, not by any other identifying information. If all eligibility criteria are met, the patient is enrolled in the study. Those who do not meet study eligibility criteria will be given local resources for the evaluation and treatment of sleep problems.

**Fig. 4:**
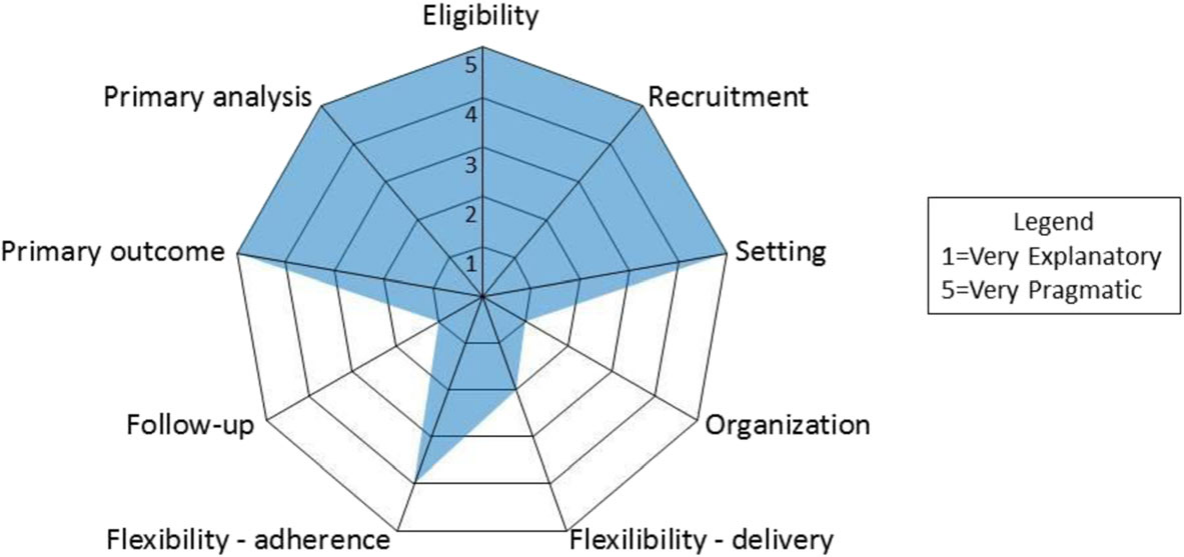
Pragmatic explanatory continuum indicator summary (PRECIS) for HUSH trial

### Baseline assessment (Fig. 3)

Enrolled participants are provided with instructions for completing baseline study instruments described below. These assessments are accessed via a secure Internet interface. The estimated total time for completion of self-report assessments, exclusive of the sleep wake diary, is 45–60 minutes. Except as otherwise indicated, the same assessments are used at the 9-week and 6- and 12-month evaluations. Objective baseline data are also collected from the EHR, as noted above.

Following completion of the baseline assessment, study staff send the BP monitor and the Apnea Link Plus unit with written instructions and web links to demonstrate their use. Participants then complete a one-week sleep diary and HBPM and a one-night ApneaLink Plus home sleep apnea test (Fig. 3). Participants keep the BP monitor for reporting at the 9-week and 6- and 12-month evaluations.

### Randomization

Eligible patients are randomized to one of three interventions, namely BBTI, SHUTi, or EUC, in a 2:2:1 ratio. We are randomly assigning 625 patients to intervention arms using stratified permuted/block design, with random block sizes of 5 and 10. Stratification by sex and age (18–40, 41–60, > 60) is employed because insomnia and HTN prevalence vary by these variables. Internet literacy may also vary by age. The randomization list is derived from computer-generated random numbers programmed by a study statistician using R version 3.2.2 statistical software. The randomization table is stored in an SQL server. After study staff determine that a participant is eligible, the data manager enters them into the randomization allocation sequence. A query is run to determine the next cell, stratified on age and sex, and the randomization sequence within that stratum; the query then assigns the participant to the appropriate cell. The allocation concealment is preserved by showing only the assignment for the participant being randomized. Subsequent allocations are not known to research staff responsible for recruiting. Study therapists provide care only in the BBTI arm, so they are not blinded to randomization. All outcome assessments are completed via self-report direct data entry through the internet to the secure research database in the Department of Psychiatry; thus, study staff with knowledge of randomization will not bias measurement of outcome data.

### Study interventions

The investigators have developed novel treatment strategies for insomnia with the goal of dissemination into routine care settings. Table 2 summarizes their key components. The two active interventions share several features, but also differ in important ways. BBTI involves a human interventionist, but the treatment is very brief. SHUTi is a self-guided, fully-automated web-based program without human interaction, but includes a larger number of modules. Adherence to each intervention is monitored by logging the dates of interventionist contact (for BBTI), core module completion (for SHUTi), or viewing of Emmi Solutions video (for EUC). Study participants are not prohibited from receiving any other care or treatment while participating in this study, and they are encouraged to follow-up with their primary care physician after the end of the study. At the start of the intervention, participants in BBTI and SHUTi receive a brief report that shows their baseline BP and sleep data. Participants in EUC receive this report, with additional information related to measures of functioning in other areas (e.g., anxiety, fatigue, pain, etc.). Each patient’s physician also receives a copy of this individualized report, which includes the baseline BP and sleep data.

**Table 2:** Intervention components

Intervention component	Intervention		
	BBTI	SHUTi	Usual care
Practices			
EpicCare RRA system to enroll study patients	•	•	•
Education on recognition/treatment of insomnia	•	•	•
Health data collected via electronic health record (blood pressure, weight, medications)	•	•	•
Physicians			
Education on insomnia; how to refer participants via RRA; interventions	•	•	•
Informed about patient diagnoses and treatment assignment	•	•	•
Receive report on patient sleep	•	•	
Participants			
Informed of insomnia diagnosis and randomization	•	•	•
Receive instructions on how to access intervention	•	•	•
Interventionist contact via telephone, web	•		
Web-based education and therapy modules		•	
Complete outcomes assessments at 9 weeks, 6 months, 12 months	•	•	•
Screening and assessments			
Telephone screening and on-line informed consent	•	•	•
Baseline and follow-up assessments of patient-reported outcomes completed on-line	•	•	•
Home blood pressure monitoring	•	•	•
Interventions			
Emmi educational video viewed on-line			•
Interventionist contact via telephone, web (4 total) for individualized behavioral sleep prescription	•		
Self-guided, web-based educational and therapy modules (6 total) with individualized instruction		•	
Feedback on initial assessment, UPMC treatment resources, and educational materials			•

*BBTI* brief behavioral treatment of insomnia, *RRA* research recruitment alert, *SHUTi* sleep healthy using the internet, *UPMC* University of Pittsburgh Medical Center

#### BBTI

BBTI is an abbreviated form of insomnia treatment appropriate for primary care settings. In contrast to traditional CBT-I, BBTI is administered in a single session and three brief follow-up sessions. Sessions are generally held weekly. Live interventionist contact is provided by telephone and/or Health Insurance Portability and Accountability Act-compliant web conferencing rather than in person. BBTI is delivered in individual sessions using a PowerPoint workbook. BBTI distills efficacious behavioral strategies for treating insomnia, including stimulus control [56] and sleep restriction [57, 58]. The treatment emphasizes sleep education and four behavioral recommendations, which are (1) reduce your time in bed; (2) get up at the same time every day of the week, regardless of how long you slept; (3) do not go to bed unless you are sleepy; and (4) do not stay in bed unless you are asleep. An initial trial of BBTI demonstrated significant improvement in self-reported and actigraphic sleep measures [33]. Patients are given a specific “prescription” of recommended sleep times and record their times in an online sleep diary via a secure web interface. Diary information gauges progress and tailors treatment.

#### SHUTi

SHUTi is a self-guided, automated, interactive, and tailored web-based program modeled on the primary tenets of CBT-I, namely sleep restriction, stimulus control, cognitive restructuring, sleep hygiene, and relapse prevention. Intervention content is metered out over time through six “Cores,” which are generally completed weekly. Users obtain access to a new Core based on a time and event-based schedule (e.g., 7 days after completion of previous Core). This schedule is consistent with recommendations from the American Academy of Sleep Medicine deeming 6–8 sessions an ‘*adequate treatment exposure*’ [13]. SHUTi uses online sleep diaries to track progress and to tailor treatment (e.g., assign a “sleep restriction” window). Each Core acts as an online analog for the weekly sessions of traditional CBT-I, and follows the same structure, including (1) Core objectives (what will be learned and why it is important); (2) review of previous week’s homework and sleep diary data; (3) new intervention material; (4) assignment of homework (treatment strategies for the coming week); and (5) summary of the Core’s main points. Intervention content is enhanced through interactive features including personalized goal-setting, graphical feedback based on participant-specific symptoms, animations/illustrations, quizzes to test user knowledge, patient vignettes, and video-based expert explanation. Automated emails encourage program adherence. After completing the post-assessment end of study battery and sleep diaries, individuals have continued access to the online program for 1 year. In an initial efficacy trial, SHUTi users showed significant improvements in sleep outcomes and insomnia severity compared to a control group. After treatment, 73% of SHUTi users had “no insomnia” vs. none of the controls, and significant improvements were also found in mood, fatigue, and quality of life [30, 59].

#### EUC

In addition to the report of baseline and screening data described above, participants in EUC also receive a listing of publically-available educational resources (websites, books) and contact information for treatment resources, as well as a link to a 25 minute animated online video developed by Emmi Solutions in consultation with two of the investigators (DJB, PJS). The video includes information about sleep, factors that help to regulate sleep, and habits that help and hurt sleep, with some interactive features, such as selecting a problem area, goal-setting, and self-efficacy assessment. After completing the post-assessment battery and sleep diaries at the 12-month follow-up, individuals randomized to EUC will subsequently have free access to SHUTi if requested.

### Follow-up evaluations

Participants in each of the three intervention conditions complete follow-up assessments at 9 weeks and 6 and 12 months following the start of the intervention. The 9-week follow-up serves as the primary outcome point for the primary study outcome measure of self-reported sleep. The 6-month follow-up serves as the primary outcome point for secondary outcomes. We promote participant retention and completion of follow-up assessments via periodic email reminders sent to participants and a quarterly participant newsletter.

### Instruments and measures

We have included validated measures intended to assess inclusion/exclusion criteria, and outcomes of interest. Information on each measure can be found below, and in the references cited.

#### Screening measures

Screening instruments are listed in Fig. 3. Insomnia diagnosis is verified during the web-based or telephone screening, using structured questions based on DSM-5 diagnosis for insomnia disorder. Insomnia of at least mild severity (Insomnia Severity Index > 7) is required at baseline to observe treatment-related changes in insomnia [60]. Other sleep disorders are also evaluated using a locally-developed screening questionnaire for sleep disorders. Likelihood of having sleep apnea is evaluated with the self-report Multivariable Apnea Prediction Questionnaire [61], and home sleep apnea testing is completed using the Apnea Link Plus or Apnea Link Air device. Participants complete a locally-developed questionnaire regarding sleep quality immediately after the home sleep apnea test night. All participants are given a report of the findings of their apnea study, with a key for how to interpret the report. Those with an Apnea Hypopnea Index score of between 15 and 50 are given a recommendation and resources for further evaluation and treatment; this information is also provided to the primary care physician. Participants are only excluded if their Apnea Hypopnea Index score is 50 or above. Locally-developed questions assess participant demographics, availability of Internet access, literacy, plans for receiving medical care over the next year, medical history, and history of bipolar disorder or psychosis. Depressive symptoms, anxiety, substance use, and suicidal ideation are assessed with measures listed in Table 1 [62–64].

#### Outcome measures (Fig. 4)

##### Sleep

The primary outcome measure is the Sleep Disturbance scale from the Patient Reported Outcome Measurement Information System (PROMIS) [65, 66]. PROMIS measures are well validated, follow a common format, and are administered in an adaptive testing format to minimize participant burden. We also collect the PROMIS Sleep-Related Impairment scale and the Insomnia Severity Index [60]. Finally, we evaluate sleep parameters daily using an on-line version of the Consensus Sleep Diary [67]. Daily sleep diaries, which take less than 5 minutes per day, are a “gold standard” outcome measure in insomnia treatment studies [67, 68].

##### Health indicators

HBPM is the primary measure of BP control. All participants are given an Omron BP7 [34] BP Monitor. HBPM is accurate when compared to ambulatory BP monitoring [69, 70], is more strongly associated with cardiovascular and cerebrovascular risk, target organ damage, and mortality than office BP readings [71–74], and is recommended as a routine component of BP measurement in patients with known or suspected HTN [70, 71, 75–78]. Participants obtain three BP readings in the seated position, with the arm supported at heart level [71, 77]. Readings are obtained twice per day, in the morning soon after awakening, and at night before going to bed, for 7 days. BP values observed on the HBPM are entered by the participant into the online sleep diary, and are collected electronically. Outcome measures include weekly average values for morning (primary outcome) and evening (secondary) BP (Fig. 3). If participants do not enter these values at each follow-up assessment, gentle prompts are sent to the participant to serve as reminders to complete this assessment. We also utilize the BP assessed during the patient’s office visit at which the research referral was made. BMI is a second objective measure of health in this study, collected via self-report during the consent assessment. Self-reported health-related functioning and quality of life are assessed with the RAND 12-item Health Status Survey [79].

##### Healthcare costs, utilization, and safety

Healthcare utilization and medical costs over the past 3 months are measured with a modification of a previously developed healthcare cost questionnaire [80]. The Cumulative Illness Rating Scale [81] is used to measure medical burden. Patients complete a modified version of the Frequency, Intensity, and Burden of Side Effects Rating [82] to determine symptoms that may be related to insomnia treatment. Patients also complete a locally-developed rating form that assesses the frequency of falls and their potential relation to insomnia treatment.

##### Treatment factors

To assess patients’ expectations for improvement as a result of treatment, and their confidence in the treatment’s ability to have an impact, they complete a modified version of the Treatment Expectations Questionnaire [83]. Patients complete the locally-developed Client Satisfaction Questionnaire to evaluate their experience participating in this study, and satisfaction with the services offered. Providers also complete a locally-developed Provider Satisfaction Questionnaire that assesses the ease of referral to the study, whether their patients entered into the study, perceived improvement of their patients as a result of participating, and satisfaction with the information we sent to the providers about their patients’ progress.

##### Health literacy

A subset of items from the Tailored Educational Approaches for Consumer Health: Market Segmentation Survey [84] is completed at baseline to assess participants’ computer and Internet use, and their health literacy.

#### Explanatory variables

Additional measures are collected, as explanatory variables, at various time points (Fig. 3). Sleep measures include the Pittsburgh Sleep Quality Index [85], which is a global measure of subjective sleep quality, and the Epworth Sleepiness Scale [86], which is a measure of daytime sleepiness. Additional health indicators are collected from the EHR, including vital signs, medical problems and diagnoses, and medication names, dosages and timing. We use additional PROMIS scales to assess psychosocial health and functioning, including measures of anxiety, depression and anger, fatigue, pain behavior, pain interference, physical function, ability to participate in social roles and activities, and satisfaction with social roles and activities [65, 66]. Finally, patients complete the Patient Global Impressions severity and improvement scales (adapted from the Clinical Global Impression scale [87]) to evaluate perceived improvement as a result of treatment.

### Sample size and power analyses

BBTI and SHUTi have medium to large effect sizes (0.40–1.00) for typical sleep and other symptom-related outcomes in published studies with sample sizes of up to 40 per group [33, 59, 88]. The observed effect sizes for this study are likely to be smaller for several reasons. Firstly, we are recruiting a more complex “real-world” sample of patients from primary care settings. Second, our recruitment method does not rely on motivated prospective participants contacting the researchers. Finally, the study design does not include any in-person contact. Thus, we relied on conservative analysis methods for conducting our power analyses. For a *t* test on post-intervention sleep outcomes comparing each treatment group to EUC, and using a Bonferroni-adjusted alpha level of 2.5% due to pairwise testing (i.e., BBTI vs. EUC and SHUTi vs. EUC), sample sizes of 180/180/90 for BBTI/SHUTi/EUC provide at least 80% power to detect an effect size of 0.4. As above, our primary outcome measures include the PROMIS Sleep Disturbance and Sleep-Related Impairment scales, the Insomnia Severity Index, and sleep parameters gathered from the Consensus Sleep Diary. Since data will be analyzed using linear mixed models, we expect to have higher power or detect smaller effect sizes. These sample sizes also provide at least 80% power to detect effect sizes that have been reported for depression and anxiety outcomes (0.4–0.8) and quality of life (0.91–1.16) [33, 59, 88]. For the exploratory aims, we will have adequate power to detect effect sizes as small as 0.3 in comparing BBTI and SHUTi given n = 200 per group. To account for up to 20% dropout rate, we propose to enroll 625 participants (BBTI = 250, SHUTi = 250, EUC = 125).

### Data management

We utilize an Internet-based, paperless data management system developed by the Department of Psychiatry at the University of Pittsburgh and UPMC. Study staff who conduct initial screenings record subject responses directly into a Microsoft SQL Server database using extensive data integrity checks. Subjects also complete measures online for direct data entry via a password-protected site. Data collection is conducted via secure, encrypted connections with data stored on a secure server at UPMC. Web sites are “insert-only,” eliminating any possibility that existing data can be retrieved from the databases. We protect confidentiality by using computer systems that are protected by the University of Pittsburgh and UPMC, which require passwords. The database servers are securely behind the UPMC firewall with permissions granted on a strict as-needed basis to members of the research team. All connections to systems inside the firewall are encrypted using a 128-bit SSL protocol. The web site for participant data entry is fully encrypted, does not utilize caching or cookies, and is strictly a one-way connection. Participant responses are inserted into the database without any permissions to select or update existing information. Participants are sent emails via UPMC’s secure server. Participants access their questionnaires via a secure link, and login with a study ID and password. Any paper records that could identify a participant are stored in locked file cabinets, and all electronic records are stored in password-protected files.

Participants in the SHUTi condition complete online sleep diaries during the course of the intervention. These diaries are maintained in the Research Infrastructure Containing E-interventions system that also hosts SHUTi at the University of Virginia. No identifying information is maintained in the servers. Electronic communications with participants are routed through UPMC’s email messaging system to maintain participant confidentiality.

### Statistical analysis

We hypothesize that BBTI and SHUTi will be superior to EUC on patient-reported symptoms (sleep, depression, anxiety, and fatigue) at 9 weeks, 6 months, and 12 months, and on health indicators (HBPM, hypnotic use, quality of life, and healthcare utilization and costs) at 6 and 12 months. The primary measure of sleep is the PROMIS sleep disturbance scale. We also hypothesize that BBTI and SHUTi will have superior measures of patient and provider level satisfaction as compared to EUC. All analyses for intervention group comparisons will use an intention-to-treat approach, in which all participants are analyzed based on the intervention to which they were randomized, regardless of intervention receipt. Ignorable missing data, which includes missing-completely-at-random and missing-at-random data, can validly be analyzed using our primary analytical methods, namely linear mixed or generalized linear mixed models for longitudinal data. Patient characteristics will be compared between completers and non-completers. Variables found to be different between these two groups will be added to the mixed models in adjusted analyses. Effects will be examined unadjusted and adjusted for stratification variables.

Analyses will use linear mixed models with fixed treatment group, time, and group*time interaction effects, and random participant intercepts. We will include random intercepts to account for within-participant correlations due to repeated measures over time. We will first assess short-term effects in each treatment group compared to EUC, using data from the 9-week follow-up. To test whether improvements in outcomes are sustained, we will expand the model to include 6 and 12 month time points. We will use similar linear mixed models in analyses of secondary outcomes (e.g., depression). For categorical outcomes, generalized linear mixed models with logit link will be used, following analysis steps described for continuous outcomes.

### Data monitoring

A four-member DSMB has been established. DSMB members have expertise in sleep medicine, insomnia intervention research, clinical trials, and biostatistics. The board meets at least annually by teleconference, and receives recruitment and adverse event updates at least quarterly. The DSMB is responsible for monitoring the overall progress and conduct of the study, the safety of the participants, and reviewing adverse events. The board will also serve as a reporting body to both the study sponsor (National Heart, Lung, and Blood Institute) and the University of Pittsburgh institutional review board (IRB). This board has been approved by the study sponsor, which also receives reports of each DSMB meeting. Adverse events are monitored by spontaneous reporting by participants and questionnaire-based inquiry using side effects questionnaires, administered at the 9-week, 6-month, and 12-month assessments. The study coordinator reviews reported events with participants. Qualifying events are reported to the University of Pittsburgh IRB, and severe adverse events are reported to members of the DSMB immediately via email. All adverse events are reviewed at regularly scheduled DSMB meetings. Changes to the protocol require approval by the University of Pittsburgh IRB and are reported to the DSMB.

### Data sharing

After study completion, results will be made available through publication in peer-reviewed journals and conference presentations. Results will be communicated to participants and referring providers via newsletters. The final dataset will be made available per our data sharing plan (additional information available upon request), which adheres to the NIH Statement on Sharing Research Data and the NIH Grants Policy on Availability of Research Results: Publications, Intellectual Property Rights, Sharing Biomedical Research Resources and Sharing Model Organisms for Biomedical Research. All investigators of the current study will have access to the final dataset.

### Assessment of pragmatic nature of study design

The National Heart, Lung, and Blood Institute program supporting this study is specifically intended to support low-cost, pragmatic [89], randomized controlled trials of interventions in existing clinical practice settings. To measure the pragmatic nature of this and other similar trials, the PRagmatic Explanatory Continuum Indicator Summary (PRECIS-2) toolkit was applied to the trial design (https://www.precis-2.org [90, 91]). While the toolkit was originally created to support the design of pragmatic trials across various domains, it is also a useful framework for analyzing the pragmatic nature of these trials post-hoc. The PRECIS-2 includes nine domains: (1) eligibility of participants; (2) recruitment of participants; (3) trial setting; (4) organizational expertise and resources needed to deliver the intervention; (5) flexibility in intervention delivery; (6) flexibility in adherence to the intervention; (7) follow-up of participants; (8) primary outcome relevant to participants; and (9) inclusion of all data in the primary analysis. A score is given to each domain using a five-point Likert scale (1 – very explanatory (basic and mechanism focused) to 5 – very pragmatic (applied and widely applicable), with 3 indicating that a trial is equally pragmatic and explanatory) [91]. These domains may be depicted visually on a radial graph for easy interpretation of the overall and domain-specific level of trial pragmatism. While there are absolute scores for each domain, the scoring may also be used for comparison to another trial to determine the level of similarity across trials on various measures of pragmatism.

Consensus ratings for this trial, and other similar trials funded by the same mechanism (RFA-HL-14-019), were completed last year by investigators of the six funded trials. Figure 4 indicates that this trial was rated as very pragmatic (score of 5) with regard to recruitment, setting, primary outcome, and primary analysis, and pragmatic (score of 4) on eligibility and adherence flexibility. On the other hand, the trial was rated as very explanatory (score of 1) on follow-up of participants, explanatory (score of 2) on organizational resources needed to deliver the intervention, and equally pragmatic and explanatory (score of 3) on flexibility in intervention delivery. As the rating of ‘very explanatory’ suggests, follow-up of participants in this study involves repeated measurement using multiple scales. As such, we have taken steps to increase interest and adherence, including (1) educating patients at study enrollment about the rationale behind the assessments; (2) sending quarterly newsletters to reinforce the importance of the study and our interest in study participants; and (3) sending reminder emails and text messages to encourage adherence at follow-up time points.

## Discussion

This study addresses a critical research gap related to insomnia treatment. While insomnia is one of the most common problems seen in primary care, safe and effective behavioral treatments for insomnia are not widely available. We are conducting a pragmatic clinical trial to test two types of behavioral treatment for insomnia versus an EUC condition. Primary outcomes focus on patient-reported measures of sleep disturbance, with a secondary outcome focusing on HTN.

The study has several strengths, including the low cost and durability of the interventions demonstrated in past trials. The estimated cost for 3 hours of social worker time (sufficient for the four sessions of BBTI) is US$90–126. The cost for a 16-week course of SHUTi is US$129. By comparison, the approximate retail price of generic zolpidem 5 mg is US$19.99 per month, or US$239.88 per year [92]. Importantly, the effects of CBT-I are more durable than those of medications [23, 24]. The trial is pragmatic in that it addresses a common clinical problem using interventions that have high potential for dissemination in real-world settings. Therapists (nurses, health educators, social workers) can be trained within a few hours to deliver BBTI, and can reach a large number of patients when the intervention is centralized by telephone/web. Likewise, SHUTi is available to any patient with Internet access. The study also leverages an academic medical center with more than 120 clinical practices that share a common EHR within a large, integrated healthcare delivery and finance system. The trial uses EHR RRAs to recruit a sample of real-world patients with INS plus HTN and to collect key health-related outcome measures. Finally, the trial emphasizes outcomes relevant to patients, providers, and health systems. The aims focus on rigorously validated patient-reported outcome measures (e.g., PROMIS measures; Fig. 4), on health indicators (e.g., health-related quality of life, HBPM) relevant to patients and providers, and on healthcare utilization and cost data relevant to healthcare systems.

Many of these advantages reflect the pragmatic nature of the trial, as depicted in the PRECIS-2 radial graph (Fig. 4) and described above. While this trial is highly pragmatic with respect to recruitment, use of existing practice settings, and relevant primary outcomes, it is less pragmatic in other respects such as in the use of follow-up methods that are not a routine part of clinical care. The less pragmatic aspects of HUSH result in part from the fact that no type of behavioral treatment of insomnia is currently integrated into practice settings at UPMC.

The current study also has some potential limitations. Participants must have access to a computer and be sufficiently comfortable and adept with using one to be enrolled. Computer and Internet access may vary by socioeconomic status and/or race. Thus, we will need to examine whether our sample is representative of those typically seen in primary care practices. Our assessments and interventions are not optimized for completion on tablets or smartphones, which may further limit access. Although we have attempted to use broad inclusion and narrow exclusion criteria to enhance generalizability, we require a current HTN diagnosis, and exclude participants for severe or untreated mental and physical disorders, which may limit generalizability. We focus recruitment on family practice and internal medicine practices, which may also limit generalizability. However, the rate of insomnia is higher in primary care patients than in the general population, suggesting that primary care is an excellent setting to screen for insomnia [49]. We also include participants who are currently taking hypnotic medications because we believe that exclusion of such participants or the requirement of medication discontinuation would hinder both the pragmatic nature of the trial and its generalizability.

Insomnia disorder is prevalent and important to patients, and has serious consequences and costs. The strengths of this trial ensure that our research will have sustained impact on how sleep treatments are scaled and delivered in real-world clinical settings. Improving the sleep health of primary care patients could improve their overall health and represent a step toward improved population health [93].

## Trial status

This trial remains ongoing with active recruitment at the time of manuscript submission.

## Additional file

Additional file 1: SPIRIT_Fillable-HUSH_Levenson. This file contains the required Standard Protocol Items: Recommendations for Interventional Trials (SPIRIT) checklist for the Hypertension with Unsatisfactory Sleep Health (HUSH) trial. (DOC 122 kb)

## Abbreviations

BBTI: brief behavioral treatment for insomnia
BMI: body mass index
CBT-I: cognitive behavioral therapy for insomnia
DSM-5: Diagnostic and Statistical Manual of Mental Disorders (5th edition)
DSMB: Data Safety Monitoring Board
EHR: electronic health record
EUC: enhanced usual care
HBPM: home blood pressure monitoring
HTN: hypertension
HUSH: hypertension with unsatisfactory sleep health
INS: insomnia
IRB: institutional review board
PRECIS: pragmatic explanatory continuum indicator summary
PROMIS: patient reported outcome measurement information system
RRA: research recruitment alert
SHUTi: sleep healthy using the internet
UPMC: University of Pittsburgh Medical Center
